# Ion adsorption and hydration forces: a comparison of crystalline mica *vs.* amorphous silica surfaces†

**DOI:** 10.1039/d3fd00049d

**Published:** 2023-03-17

**Authors:** Igor Siretanu, Simone R. van Lin, Frieder Mugele

**Affiliations:** a Physics of Complex Fluids Group and MESA+ Institute, Faculty of Science and Technology, University of Twente PO Box 217 7500 AE Enschede The Netherlands i.siretanu@utwente.nl f.mugele@utwente.nl

## Abstract

Hydration forces are ubiquitous in nature and technology. Yet, the characterization of interfacial hydration structures and their dependence on the nature of the substrate and the presence of ions have remained challenging and controversial. We present a systematic study using dynamic Atomic Force Microscopy of hydration forces on mica surfaces and amorphous silica surfaces in aqueous electrolytes containing chloride salts of various alkali and earth alkaline cations of variable concentrations at pH values between 3 and 9. Our measurements with ultra-sharp AFM tips demonstrate the presence of both oscillatory and monotonically decaying hydration forces of very similar strength on both atomically smooth mica and amorphous silica surfaces with a roughness comparable to the size of a water molecule. The characteristic range of the forces is approximately 1 nm, independent of the fluid composition. Force oscillations are consistent with the size of water molecules for all conditions investigated. Weakly hydrated Cs^+^ ions are the only exception: they disrupt the oscillatory hydration structure and induce attractive monotonic hydration forces. On silica, force oscillations are also smeared out if the size of the AFM tip exceeds the characteristic lateral scale of the surface roughness. The observation of attractive monotonic hydration forces for asymmetric systems suggests opportunities to probe water polarization.

## Abstract 2

A systematic study using dynamic Atomic Force Microscopy of hydration forces on mica surfaces and amorphous silica surfaces in aqueous electrolytes containing chloride salts of various alkali and earth alkaline cations of variable concentrations.

## Graphical abstract

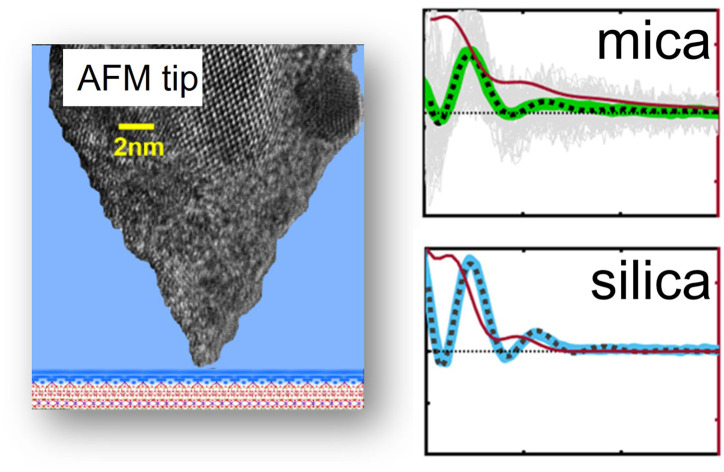

## Introduction

The interfacial water structure and the associated short range hydration forces have long been recognized as essential for many phenomena and processes in nature and technology including the stability of colloidal systems,^1,2^ the assembly of soft biological and non-biological matter on molecular and supramolecular scales,^3,4^ wetting,^5^ lubrication,^6^ and the swelling of clay induced by nanoconfined water.^7^ More recently, there has been increasing evidence for the relevancy of hydration effects in catalysis, including in particular electrocatalytic water splitting.^8^ Moreover, adsorbed water at mineral surfaces is relevant for CO_2_ sorption and fixation.^9^ Water is unique as a fluid because of its very large dipole moment and its ability to form hydrogen bonds. This leads to a particularly strong coupling between the solvent, dissolved ions and charged or hydrogen bond-forming sites on a surface.^10^ On the colloidal scale of ≈1 nm and beyond, the structure and composition of interfacial water is rather well described by classical continuum Derjaguin–Landau–Verwey–Overbeek (DLVO) theory.^11^ However, it is ‘the last nanometer’ where DLVO theory fails that ultimately controls the formation of mechanical contact and chemical bonds and determines the surface charge experienced on the continuum colloidal scale.

Classically, the structure of this compact part of the electric double layer has been described by allowing ions to adsorb with specific adsorption energies in one or more planes above the surface depending on their size and degree of hydration, while the ambient solvent is considered as a passive continuum dielectric. While often empirically successful, such a description is too simplistic to capture the complexity of discrete ions and discrete water molecules interacting with discrete sites on the solid surface and depends strongly on *ad hoc* assumptions regarding the kind of surface speciation reactions that are taken into account. For instance, Pashley and Israelachvili extracted in their seminal studies of DLVO-forces in aqueous electrolytes values for the equilibrium constants for the adsorption of various cations to mica surfaces assuming cation and hydronium adsorption as possible surface speciation reactions.^12,13^ By now, it is rather clear from X-ray surface diffraction measurements,^14,15^ AFM,^16^ and Molecular Dynamics (MD) simulations^17,18^ that the negative intrinsic surface charge of mica is often overscreened by adsorbing alkali cations. The positive excess charge is then compensated by co-ions in a second layer leading to a net negative surface charge density, as seen from the colloidal perspective. Force measurements on the colloidal scale are only sensitive to the diffuse layer charge (or potential) and therefore provide only indirect information on the compact part of the EDL. Any microscopic structure leading to the same diffuse layer charge provides an equally acceptable model for the DLVO forces.^19^

Non-DLVO hydration forces with a strongly oscillatory character reflecting the discreteness of water molecules were reported for the first time in the 1980s in the pioneering work by Pashley and Israelachvili^12,13,20–22^ between two atomically smooth mica surfaces. The forces displayed a range of 1–2 nm and a periodicity corresponding to the size of a water molecule. In the meantime, advances in experimental techniques such as X-ray reflectometry and surface diffraction,^23,24^ non-linear optical spectroscopies,^25^ and Atomic Force Microscopy (AFM)^26–28^ along with powerful Molecular Dynamics (MD),^17,18^ Monte Carlo (MC)^29^ and Density Functional Theory (DFT)^30^ simulations have produced detailed complementary insights into the structure and dynamics of interfacial water. Especially, recent advances in the field of AFM, including so-called three-dimensional atomic force microscopy (3D-AFM)^26,31,32^ have revealed detailed structural information including preferred binding sites for water and ions on specific model surfaces, namely mica and the (01-04) cleavage plane of calcite. Comparison of the experiments to MD simulations led to the insight that the forces experienced by ultra-sharp AFM tips with a radius of 1–2 nm often closely resemble the force that can be derived from the potential of mean force experienced by a single water molecule at an isolated substrate–electrolyte interface. This so-called solvent–tip approximation has been used successfully to describe atomically resolved images of the crystalline lattice, atomic scale defects, as well as the hydration structure of the surface.^26,31,33^ Kilpatrick *et al.*^27^ as well as van Lin *et al.*^34^ demonstrated that the hydration forces on mica can be described for a wide range of fluid compositions by a superposition of an oscillatory and a monotonically decaying hydration force. In particular, van Lin *et al.*^34^ found that the strength of the monotonic hydration force gradually decreases with the decreasing bulk hydration energy of ions, leading to a transition from an overall repulsive (Li^+^, Na^+^) to an attractive (Rb^+^, Cs^+^) monotonic hydration force. While the oscillatory part, which is related to intrinsic structured water layers at the mica surface, is hardly affected by the presence of strongly hydrated cations (Li^+^, Na^+^), it becomes disrupted by the presence of weakly hydrated cations (Rb^+^, Cs^+^). This suppression of oscillatory force was consistent with the water structure in MD simulations if interpreted in terms of the solvent–tip approximation.^18,34^

While oscillatory hydration forces have also been reported for a few other atomically smooth surfaces^17,31,35,36^ data on other types of surfaces including the very abundant and important silica surfaces are rather scarce and inconsistent. A few experimental studies from the 1980's address the topic of hydration forces between amorphous silica surfaces from the experimental perspective, as reviewed by Valle-Delgado *et al.*^37^ Several authors reported the existence of monotonically decaying repulsive non-DLVO forces with a range of less than 2 nm by means of surface force apparatus or colloidal atomic force microscopy. However, there is no consensus regarding the origin and the dependence of this force on fluid composition (ion concentration, type of ions, pH). For instance Horn *et al.*^38^ measured the monotonic short-range repulsive hydration forces in NaCl solutions between silica surfaces. The forces were independent of the salt concentration and therefore interpreted as intrinsic to silica surfaces. Rabinovich *et al.*^39^ and Peschel *et al.*^40^ found similar results and attributed this “additional” component of force by subtracting the DLVO theory to a “hydration repulsion” similar to the one between mica surfaces and lipid layers. In line with a widespread notion, the authors attributed the absence of an oscillatory component to the hydration force to the finite roughness of the substrates that would smear out any oscillations, as also discussed by Israelachvili and Wennerström.^1^ Alternatively, it was proposed in some earlier studies that the absence of oscillatory hydration forces and the appearance of a monotonically decaying repulsive force would be caused by a steric repulsion between short ‘hairy structures’ of silica and silicates protruding a few Angstroms from the silica surface.^41,42^ Colloidal probe AFM measurements by Ducker *et al.*^43,44^ reported a short range monotonic force that seemed to increase with surface charge in contrast to Grabbe and Horn *et al.*^38,45^ who did not find any systematic dependence on salt concentration and pH. Later, Chapel^46^ measured forces between two pyrogenic silica sheets immersed in monovalent electrolytes (CsCl, KCl, NaCl, and LiCl). Contrary to the previous results and to observations on mica, his results showed that the strength and the range of the hydration force decrease with increasing the degree of hydration of the counter ion. At present, to our knowledge the recent work by Klaassen *et al.*^36^ is the only experimental study that reports oscillatory hydration forces on silica surfaces, which, however, were observed only for a relatively small fraction of the approach curves. It is thus unclear whether oscillatory hydration forces on these amorphous surfaces are common or whether they are usually averaged out by the unavoidable finite surface roughness or the heterogeneous interactions with the different conformation of surface oxide and silanol groups. Similarly, it is unclear how the more routinely observed monotonically decaying hydration forces depend on the surface charge and how they are affected by the presence of ions.

To address these questions, we extend in this work our previous high resolution AFM spectroscopy study of hydration forces on atomically smooth mica surfaces to amorphous silica surfaces and include both monovalent alkali and divalent earth alkaline chloride salts at variable pH. To enable a direct comparison between the two types of surfaces, the data shown in this work are recorded with the same AFM tip in the same electrolyte after each other on mica and on silica, unless otherwise noted. Overall, our measurements with ultra-sharp AFM tips display a remarkable similarity between mica and silica substrates. For all fluid compositions, we observe both oscillatory and monotonically decaying hydration forces independent of pH and type and concentration of cations in the solution. The only exception are Cs^+^ ions, which destroy the oscillatory hydration structure on silica in the same way as reported earlier for mica. Thus, neither the differences in surface roughness nor the charge density and charge distribution have a significant impact on the hydration forces of these two model systems. This suggests that the observed hydration forces are dominated by local hydrogen bonding of water molecules with pH-independent binding sites.

## Methods and materials

### Amplitude modulation (AM)-AFM spectroscopy

All AFM measurements are performed using an Asylum Research Cypher ES equipped with photothermal excitation. The experiments were performed at a controlled temperature of 28 °C in a closed liquid cell that allows for *in situ* fluid exchange. The cantilever was immersed in a droplet of liquid (0.2 mL) sandwiched between the substrate and the top of the cell. The fluid was exchanged using two glass syringes by injecting a new solution while completely removing the old solution. The drop volume was exchanged at least 25 times with a new fluid. Any new fluid was left to equilibrate for 30 minutes before starting the next measurements. The force spectroscopy was performed in amplitude modulation (AM) mode.^34,36,47,48^ During this procedure the amplitude and phase of the cantilever deflection were tracked as a function of *z*-piezo distance. To measure the tip–sample interactions, the cantilever was driven at a fixed frequency (*ω* ≈ 0.97*ω*_0_, where *ω*_0_ ≈ 450 kHz) by an intensity-modulated blue laser diode that was focused on the gold coated topside of the cantilever close to its base. AFM measurements were operated with an oscillation amplitude of *A* = 0.2–0.3 nm. For each amplitude– and phase–distance curve, the cantilever was ramped over 5 nm. We typically measured 100–200 individual approach curves (represented by the grey lines in the figures) for each fluid composition while keeping the tip at the same nominal position on the samples (mica or silica). We started the experiments with mica (as a model surface since the hydration forces behavior was known already^34^) in the following order of fluid composition: deionized water; pH 6, 50 mM LiCl; pH 6, 50 mM NaCl; pH 6, 50 mM KCl; pH 6, 50 mM CsCl and then again deionized water. Then the mica was exchanged by silica and the above sequence of experiment was repeated. The same results were obtained when order of samples and electrolytes was switched. To protect the shape of the tip apex, the amplitude signal was not allowed to drop below 80–90% of its free amplitude. The recorded curves were aligned to the first oscillation found in the force gradient profile. The first oscillation's maximum was set at an apparent tip–sample separation of approximately 0.25 nm. This sets the 0, where we find the linear region in the deflection data indicating the tip–substrate contact.

### Experimental preparation

The mica and silica substrates were glued with epoxy to a steel puck which was magnetically clamped to the piezo stage of the AFM. Muscovite was cleaved with adhesive tape before each experiment. As silica sample surfaces, we used a silicon wafer (1 × 1 cm) with a 30 nm thermally grown oxidized layer. The silica sample was cleaned in an ultrasonic bath for 10 min in a mixture of isopropanol, ethanol, and Millipore water (25/25/50% by volume) and subsequently rinsed with only Millipore water. Then, the substrate was air plasma cleaned (PDC-32G-2, Harrick Plasma, Ithaca, NY, USA) for 20 min. Each experiment was started by flushing the system with purified water. The electrolyte solutions were prepared by dissolving the salts (NaCl, KCl, LiCl and CsCl 99% purity) in purified water. The pH was controlled by adding either NaOH or HCl to the solution. The pH of the electrolyte solutions without adjustment is around 6. All the used chemicals were purchased from Sigma Aldrich.

### Cantilevers

Gold coated (detector side) silicon ultra-high frequency cantilevers (ARROW-UHFAuD, nanoworld) were used. The length and width were 35 μm and 42 μm, respectively. The tip has an arrow shape and the force constant (*k*), resonant frequency (*f*) and quality factor (*Q*) of the first eigenmode as determined in liquid were in the range: *k* = 1.23–3.35 N m^−1^, *f* = 600–1000 kHz and *Q* = 6. The above values are determined in purified water (Millipore, resistivity 18.2 MΩ cm). The force constant is determined by using the thermal method. Prior to use, the cantilevers are cleaned by putting them in a bath of 1 : 1 ethanol/isopropanol for 15 minutes, after which they were dried using air and placed in a plasma cleaner (PDC-32G-2, Harrick Plasma) for 20 minutes. The tip radius was determined after data collection from the HRTEM imaging, and was found to be around 2 nm for all the levers used in this study (which is an upper limit since this was measured after calibrating the tip upon being pressed into contact). Relative stiff cantilevers were used in order to prevent mechanical instabilities in the presence of attractive force gradients.

## Results

**Fig. 1:**
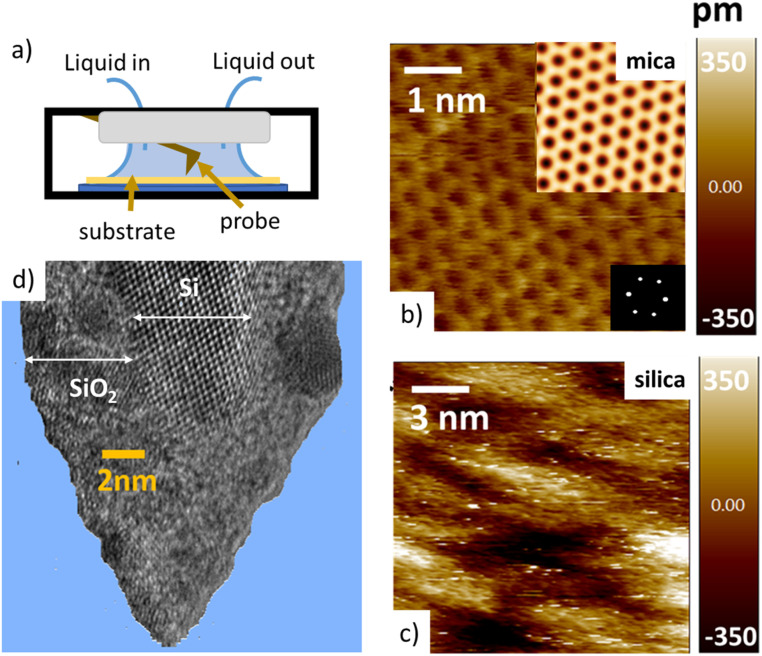
(a) Schematic view of the atomic force microscope liquid-cell. (b) and (c) typical AFM images of mica and silica surfaces. (d) High Resolution Transmission Electron Microscopy (HR-TEM) image of a silicon tip after an experiment. Note the crystalline silicon core and the surrounding amorphous silicon oxide (for clarity, the TEM image was processed and the amorphous carbon matrix surrounding the tip was removed).

**Fig. 2:**
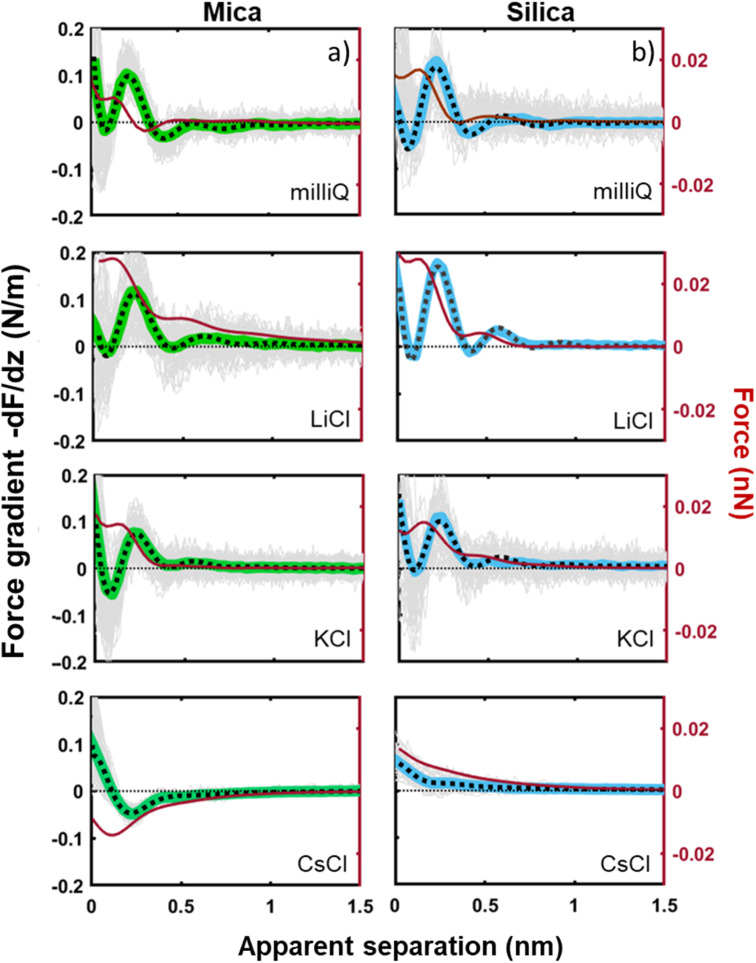
Averaged force gradient (−d*F*/d*z*; thick green lines for mica and thick blue lines for silica) and force (*F* thin dark red lines) *versus* apparent tip–sample separation measured in pure water (top panel; light grey: 180 individual force curves) and for various salt solutions at a fixed concentration of 50 mM. All the data were recorded with the same AFM tip at pH 6. Dotted lines: empirical fits to eqn (1).

**Fig. 3:**
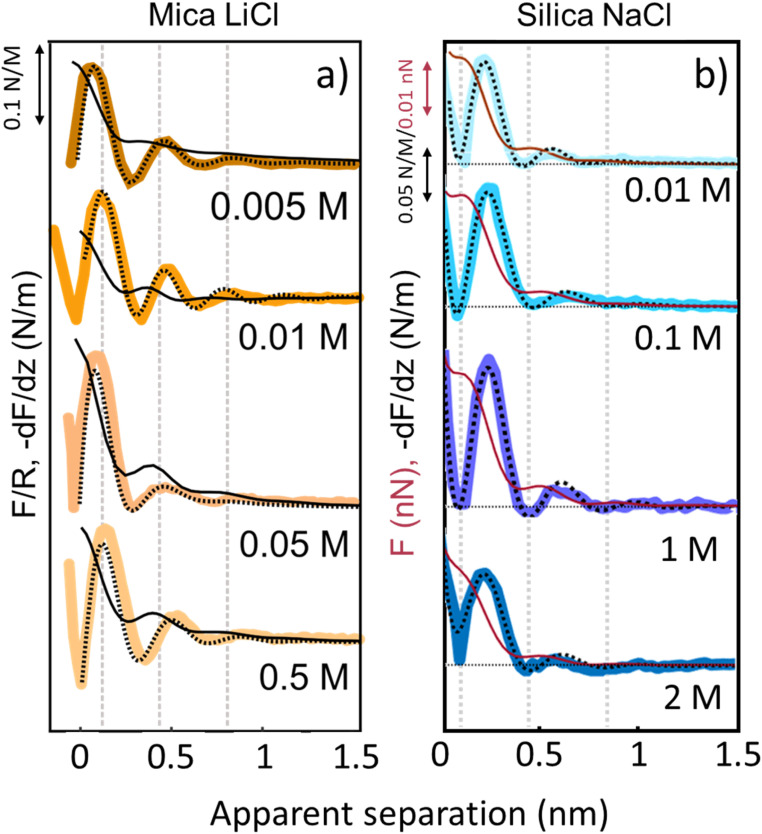
Averaged (*n* = 80) force gradient (−d*F*/d*z*; thick lines) and force (*F* thin dark red lines on silica and *F*/*R* thin black lines on mica) *versus* apparent tip–sample separation of various concentrations of LiCl on mica (a) and of NaCl on silica (b). Each salt was measured with a separate AFM tip at pH 6. Dashed lines: empirical fits to eqn (1).

**Fig. 4:**
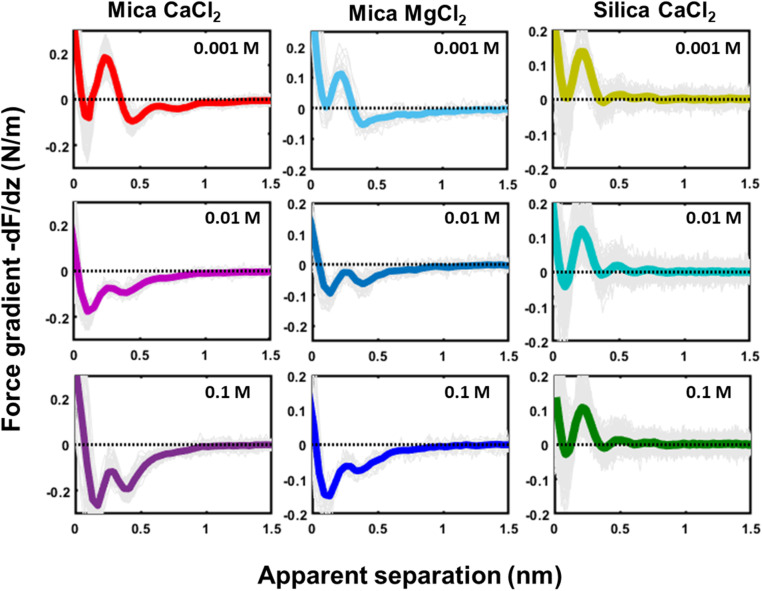
Force gradient (−d*F*/d*z* (N m^−1^)) *versus* apparent separation (nm) measured in 1, 10 and 100 mM of CaCl_2_ and MgCl_2_ on a mica substrate and of CaCl_2_ on a silica substrate. The thin gray lines are individual measured curves, the thick line is the average of these lines. The measurements at the mica and silica substrate are obtained with different tips at pH 6.

Tip–sample interaction forces are measured in non-contact amplitude modulation atomic force microscopy (AM-AFM), following well-established operation and data analysis procedures.^47–49^ During the AFM experiments, the mica and silica surfaces, together with the AFM tip–cantilever assembly are fully immersed in a drop of the aqueous salt solution under consideration (Fig. 1a). The crystalline lattice of the atomically smooth mica surfaces is easily resolved in high resolution images (Fig. 1b). The amorphous silica surface (SiO_2_) displays a somewhat higher roughness with height variations of approximately 0.3 nm, *i.e.* slightly more than the diameter of a water molecule, on a lateral scale of 2–4 nm (Fig. 1c). The characteristic lateral scale is thus substantially larger than on mica. In particular, it is larger than the typical radius of 1–2 nm of the ultra-sharp AFM tips of the present experiments (Fig. 1d). For consistent comparison among the substrates, the forces for mica and silica surfaces are measured immediately after each other using the same AFM tip and salt solutions. More than 100 force–distance approach curves (grey lines in Fig. 2 and 4) are typically recorded for each fluid composition at a rate of 1–2 approach curves per second. Slight mechanical drift of a few nm throughout the measurement time implies that force curves should be considered as originating from random locations on a surface area ranging from several up to a few tens of nm^2^. No attempt is made to reconstruct a 3D force map. Instead, the thick colored lines in Fig. 2 to 4 and 6 below report averages of the individual force gradient curves. Prior to averaging, force gradient curves are aligned along the *z*-direction such that the first (repulsive) maxima of the characteristic force oscillations are aligned. This procedure enhances the strength of the force oscillations as compared to an averaging procedure based on *e.g.* the retraction point force curve. Thin red lines show the corresponding force–distance curves obtained from the force gradient data by numerical integration.

### Dependence of hydration forces on cation species and concentration

The tip–sample interaction (force gradient and force) for mica and silica surfaces measured in deionized water and for salt solutions containing 50 mM of LiCl, KCl, and CsCl are presented in Fig. 2. The data display a number of striking aspects. First of all, the range of the forces for all systems is limited to approximately 1 nm. Neither continuum electric double layer forces nor long range van der Waals forces are detected. This is characteristic for AFM measurements with ultra-sharp tips (see *e.g.* ref. 26, 31, 32, 34, 35, 50, 51). With a typical (bare) surface charge density of −0.1*e* nm^−2^ or less for silica–electrolyte interfaces at pH 6,^52^ the surface area of the tip that probes the force is too small to carry sufficient charge to generate a measurable electrostatic force. Similarly, van der Waals forces decrease with decreasing tip size and are typically very small. As a result, the measured forces with ultrasharp AFM tips are dominated by the short-range molecular environment of the tip. This observation is the basis of the so-called solvent–tip approximation introduced earlier by Reischl and Watkins,^33^ which states that the force experienced by an ultrasharp AFM tip in water is dominated by the interaction of the outermost hydration molecule(s) of the tip with the hydration structure (and possibly adsorbed ions) at the sample surface. This implies that the tip–sample interaction force can be approximated by the gradient of the potential of mean force acting on a water molecule at an isolated substrate–electrolyte interface. This approximation proved to be successful for a variety of systems,^31,33^ including our earlier combined AFM-MD study on mica,^34^ which included the fluid compositions shown here in Fig. 2. The fact that the forces display an oscillatory character with spacing between the maxima close to the diameter of a water molecule (∼0.28 nm) then leads to the conclusion that the oscillatory forces are caused by the hydration structure of the substrate. No features are observed that would correspond to the diameter of hydrated ions or ion pairs.

The second striking observation is that the pronounced oscillations of the force gradient are observed both for pure de-ionized water and in the presence of 50 mM of LiCl and KCl. The only difference is a slight overall repulsion for LiCl consistent with our earlier results.^34^ This observation is different from early SFA^12,13,53^ and AFM^27^ measurements on mica, which argued that a finite concentration of cations, preferentially a few tens of mM of K^+^ ions (and 1 M of Ca^2+^) would be required to observe oscillatory hydration forces. Our measurements suggest that this was probably a consequence of the poorer sensitivity of those measurements that report directly forces whereas our dynamic AM-AFM measurement technique reports the force gradient as primary data, which naturally emphasizes any oscillatory behavior. In contrast to LiCl and KCl solutions, CsCl has a very strong effect on the forces. It completely eliminates the oscillatory hydration structure and induces a slight attractive interaction on mica and a slight repulsion on silica, which both decay within approximately 1 nm. In our earlier combined AFM-MD study on mica,^34^ we interpreted this observation as follows: for all salts cations adsorb to the mica surface in the simulation primarily in inner shell configuration to an extent that they compensate the intrinsic charge density of the mica surface. This leads to a cation coverage of approximately 1 nm^−2^. In the case of the smaller and more strongly hydrated Li^+^ and K^+^ ions, this coverage still leaves sufficient ‘bare’ mica directly exposed to the ambient water to keep the intrinsic hydration structure that we observe in pure water intact. Moreover, the hydration shells of the Li^+^ and K^+^ seem compatible with the intrinsic hydration structure of the substrate such that the overall hydration forces on the partly cation-covered substrate remain similar to the case of pure fluids. In contrast, the bulkier and less hydrated Cs^+^ ions severely disrupt the intrinsic hydration structure of mica. In MD simulations, this leads to a pronounced decrease in the water density close to the substrate – and a disruption of the oscillatory density profile. Applying the solvent–tip approximation, the absence of oscillations in the water density profile then leads to a non-oscillatory potential of mean force and thereby explains the absence of oscillations in the force profiles.^34^

The third and final remarkable observation from Fig. 2 is that all these observations that have been reported before for mica also hold for the very different amorphous silica surfaces: hydration forces display a strongly oscillatory character in both pure water and in the presence of small Li^+^ and K^+^ cations (and also Na^+^; see Fig. 3b). For both substrates, force oscillations disappear for the larger and weakly hydrated Cs^+^ cations. On mica, the same applies for RbCl solutions, too (data not shown; see ref. 34). Superimposed onto this oscillatory force, there is a monotonically decaying background force that is most pronounced for Li^+^ and decreases with increasing ion size. The only appreciable difference between mica and silica is that the monotonic force is attractive in CsCl solutions on mica but remains repulsive in case of silica. Fig. SI2† confirms this result for an independent second experiment with a different AFM tip of the same type. The similarity between the two substrates is not limited to the specific salt concentration of 50 mM chosen in Fig. 2. Fig. 3 demonstrates this for NaCl and LiCl solutions across a wide range of concentrations. Fig. 3 also shows that the range of the forces is independent of the salt concentration (Fig. SI4 and SI5†). This independence implies that the forces measured with these ultrasharp AFM tips are not caused by classical DLVO forces, which would display a tenfold increase in the decay length from the highest to the lowest concentration shown in this figure based on Debye screening.

To capture these qualitative trends described above in quantitative numbers, we can fit the force gradient data using an empirical function that describes the total hydration force as a superposition of an oscillatory component with an exponentially decaying amplitude and a monotonically exponentially decaying component^11,27^1
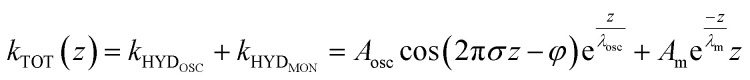
In this formula, *A*_osc_ and *A*_m_ describe the strength of the oscillatory and the monotonic hydration forces, *φ* is the phase shift, *σ* is the structural hydration layer spacing, and *λ*_osc_ and *λ*_m_ are the decay lengths of the oscillatory and monotonic forces. The fitting procedure yields separate numbers for the decay lengths of the oscillatory and the monotonic contributions of ∼0.12 ± 0.03 and ∼0.32 ± 0.06 nm on mica and ∼0.12 ± 0.02 nm and ∼0.28 ± 0.04 nm on silica, respectively. In all cases, the average distance (*σ*) between the adjacent hydration layers calculated using a periodic cosine function is 0.27 ± 0.03 nm for mica and 0.28 ± 0.04 nm for silica, consistent with the size of a water molecule. As already stated above, no appreciable dependence on the type of cation nor on the concentration is observed; except obviously for Cs^+^ ions, which suppress the force oscillations altogether. Minor variations of the amplitudes *A*_osc_ between pure water, LiCl and KCl solutions are occasionally observed but vary from tip to tip and are also sensitive to details of the fitting procedure. Consistent is the observation that *A*_m_ is positive on both substrates for de-ionized water, LiCl, and KCl solutions for all conditions with Li^+^ displaying the strongest repulsion. This confirms the results of our early study on mica, where we reported that the strength of the monotonically decaying background force follows the order of the bulk hydration energy Li^+^ ≥ Na^+^ > K^+^ > Cs^+^ (see van Lin^34^). Our results contradict earlier work of Chapel^46^ who reported the opposite order of the ion-specific effect on silica. However, those results were based on laterally averaging SFA measurements that were subject to roughness-induced effects. The present data show that the sign reversal of *A*_m_ for Cs^+^ occurs only on mica but not on silica. As we will discuss in more detail below, we attribute this sign reversal to the appearance of an attractive monotonic hydration force in the presence of Cs^+^. A detailed overview of all fit parameters is reported in Fig. SI3.†

Similar qualitative trends are also observed for solutions CaCl_2_, MgCl_2_ (Fig. 4) and BaCl_2_ (data not shown; see ref. 54). Like in the case of monovalent cations, a combination of oscillatory and monotonically decaying hydration forces is found on both substrates, with a periodicity of the force oscillations comparable to the diameter of a water molecule and a range of ∼1 nm. The force oscillations persist from the lowest to the highest concentration of Ca^2+^, Mg^2+^ and Ba^2+^ cations. This observation is worth noting because frequently cited early SFA measurements suggest that hydration forces in the presence of earth alkaline chloride salts only become oscillatory at concentrations above 1.0 M.^12^ Our measurements show that this is not the case and that both silica and mica display intrinsically oscillatory hydration forces even in pure if interrogated by a sufficiently sharp probe. Earth alkaline cations do not change this behavior (Fig. 4).

The most striking difference between mica and silica solutions containing Ca^2+^, Mg^2+^, and Ba^2+^ cations is that the monotonic hydration force always remains repulsive on SiO_2_ substrates, whereas it becomes attractive on mica, like in the case of Cs^+^ discussed above (Fig. 4). We will discuss the likely origin of this difference below.

## Discussion

### Dependence on surface roughness

**Fig. 5:**
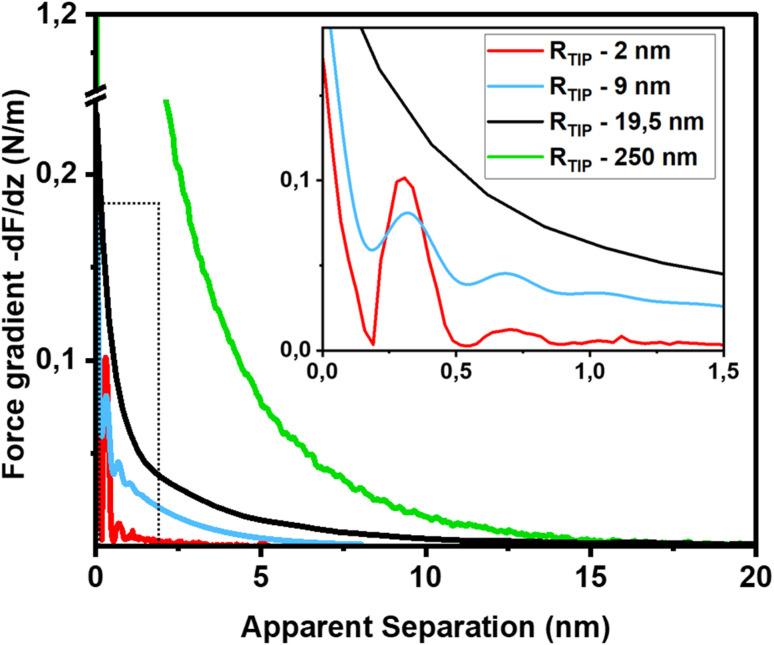
Force gradient *vs.* separation curves on smooth silica surfaces recorded with AFM tips of different size (10 mM NaCl solution; pH 6). Solid red line: same data as in Fig. 3b (tip radius (*R*_TIP_) ≈ 2 nm); blue line: from ref. 36 (tip radius: 9 nm); black line (tip radius: 19.5 nm): green line from ref. 56 (tip radius: 250 nm). The SEM and HR-TEM images of the AFM tips are shown in ESI Fig. SI6.†

The first important point of discussion is that we do actually see oscillatory hydration forces on silica in a very similar manner as on mica. This is in contrast to many earlier reports using surface force measurements with the SFA, colloidal probe AFM as well as regular (*i.e.* not too sharp) AFM tips.^12,13,38,45,47,55–57^ Our conclusion is thus that silica–water interfaces display an intrinsically oscillatory hydration structure similar to atomically smooth hydrophilic surfaces such as mica and calcite. However, since silica is not atomically smooth but displays a roughness of the order of the diameter of a water molecule, the intrinsic oscillations are averaged out if the probe is not sufficiently sharp but averages over surface regions of different local height. As shown in Fig. 1c, the characteristic lateral scale of the roughness of our wafers is of the order of several nanometers. Ultrasharp tips are thus sharp enough to avoid cancellation by this averaging effect, whereas larger ones are not. To illustrate this point, we compare in Fig. 5 force gradient curves obtained on identically treated silica surfaces for the same fluid composition but with different tip radii from ≈2 nm to 250 nm: clearly, the oscillatory hydration forces are gradually smeared out for tips of larger radius (Fig. 5 inset). At the same time, the probes become increasingly susceptible to continuum DLVO forces. This tendency becomes even more pronounced for colloidal probe tips with a radii of 100 nm and beyond, see *e.g.* ref. 56–58. To our knowledge, all previously reported systematic studies of hydration forces on silica surfaces were performed with tips with radii of 25 nm and more. (We refrain here from normalizing the interaction forces by the tip radius as commonly done in the surface forces community. The rationale for this normalization is the Derjaguin approximation, which states that the force normalized by the tip radius is proportional to the surface energy. This approximation is based on the assumption that the tip radius is large compared to the tip–sample separation, which is not fulfilled for the smallest tips.^11,59^).

The fact that silica intrinsically displays oscillatory forces with a spacing of ≈0.28 nm is strong evidence that the forces are indeed hydration forces – a conclusion that is more difficult to draw if only monotonically decaying forces are observed. It shows that silica locally behaves very similar to atomically smooth surfaces. It would be very unlikely that the monotonically decaying hydration forces that we observe in addition to the oscillatory ones originate from steric repulsion by ‘hairy’ silicate or polysilicic acid structures, as previously suggested.^1,41,42^ It would be hard to reconcile how such structures would induce fairly strong repulsive monotonically decaying forces while leaving the oscillatory part of the hydration structure intact.

### Dependence on pH and surface charge

**Fig. 6:**
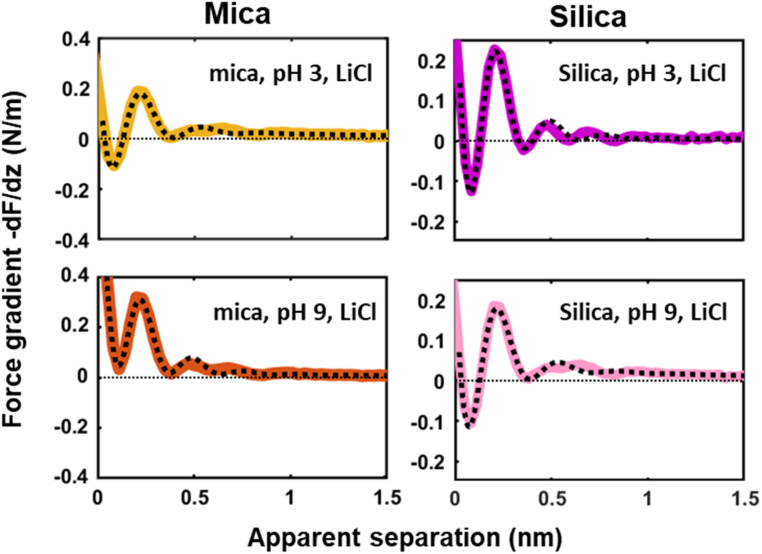
Averaged (*n* = 100) force gradient (−d*F*/d*z*; colored thick lines) *versus* apparent tip–sample separation measured in 50 mM LiCl solutions, with bulk pH values of pH 3 and 9, on a silica and mica substrate. The pH was adjusted by adding either HCl or NaOH. The data shown here were collected using the same AFM probe. The dotted lines represent the fit using eqn (1).

Next to crystallinity and roughness, the surface charge and its distribution is another key difference between mica and silica. The surface charge of mica is determined by the crystalline lattice. It has a fixed value of one negative unit charge per surface unit cell (*i.e.* −2.1*e* nm^−2^) independent of pH. This charge is distributed over a triad of three oxygen atoms bound to a tetrahedrally coordinated Al atom that substitutes a Si atom in the topmost crystalline layer. In contrast, the silica surface is intrinsically uncharged and assumes its surface charge only by pH-dependent deprotonation of silanol groups. While there are different types of silanol groups that are either isolated or partly incorporated into the Si–O (siloxane) rings of the material,^60^ the residual negative charge nevertheless essentially remains localized on the remaining deprotonated oxygen atom on the surface. The surface charge of silica vanishes at the isoelectric point pH = ≈2.5–3 and increases to ≈−0.1*e* nm^−2^ at pH 6 and further to ≈−0.94*e* nm^−2^ at pH 9 in 0.2 M solutions of LiCl, NaCl, and KCl according to macroscopic titration studies.^52,61^ Yet, despite these large variations in surface charge, the general trends of the hydration forces as probed by an ultrasharp AFM tip are almost identical on the two substrates at pH 6 (Fig. 2 and 3) and also if we decrease or increase the pH of the solution, as shown in Fig. 6. Oscillatory hydration forces are found for LiCl solutions for all pH values on both substrates; similarly, CsCl destroys the hydration forces on both substrates at all pH's investigated in the same manner (data not shown; see ref. 54). Only the sign of the monotonic forces is repulsive for silica at pH 6 and 9, while an attractive minimum is visible on mica for all pH values. This rather weak pH-dependence on silica suggests that the hydration forces are actually dominated by the interaction of the water molecules with undissociated silanol groups as well as bridging oxygen atoms in siloxane rings on the surface.^30,62,63^ Such a dominance is reasonable given the fact that even at pH 9 the majority of the silanol groups on the surface remains protonated.^19^ Moreover, most of the deprotonated groups are expected to be complexed by a cation from solution, as reported earlier for NaCl and KCl solutions.^19^ In addition to the weak pH dependence, the predominance of protonated silanol groups and bridging oxygen atoms also explains the weak dependence of the hydration forces on the presence the smaller hydrated alkali cations. These ions adsorb only weakly to silica and leave the majority of the surface area unaltered,^64^ as evidenced *e.g.* by the very weak reduction of the average silanol–water coordination number from 2.83 (pure water) to 2.67 in 200 mM NaCl.^65,66^ Consistent with earlier studies by Horn *et al.*^38,45^ and Chapel^46^ and recent MD simulations^62,65–69^ we argue that the hydration structure and force on silica primarily originates from hydrogen bonding of water molecules to undissociated silanol and siloxane groups. While the detailed configuration depends on the local bonding geometry and atomic scale surface roughness, water molecules predominantly act as donors in the H-bonds to the substrate. This mechanism – and not the weak pH-dependent surface charge – leads on the average orientation of water dipoles pointing away from the surface in the first hydration layer, as seen in SFG experiments and simulations.^62,63,68,70,71^ The ‘anchoring’ thus imposed onto the first surface-bound water layer is subsequently propagated to subsequent hydration layers, where it is eventually probed by the overlap with the hydration structure (or solvent-tip) of the AFM tip.

For the mica surface, the situation is different in the sense that the surface does carry a strong and pH-independent net surface charge, as discussed above. Nevertheless, the surface is chemically dominated by more or less partially negatively charged oxygen atoms bridging either two Si atoms or a Si and an Al atom in the lattice. Notwithstanding the difference regarding order and charge distribution, from the perspective of water molecules the mica surface thus presents a distribution of negative oxygen atoms that can act as the proton acceptor in a H-bond. Details are certainly different, but overall the first surface-binding hydration layer on mica experiences a H-bond accepting surface that is very similar to the case of silica. As a result, the first layer is anchored in a similar manner with water dipoles predominantly pointing away from the surface.^17,29,34,69,72^ One might object that the dependence on cation adsorption should be very different between the two substrates because alkali cations adsorb much more strongly on mica than on silica in order to compensate the negative intrinsic surface charge. Yet, a series of studies has shown that adsorbed alkali cations have little effect on the organization and preferential orientation of the surface-bound layer, as long as the cation is sufficiently small and strongly hydrated.^17,34^

Accepting that the organization imposed onto the directly surface-bound water layer is thus similar and assuming that the subsequent propagation of ‘hydration information’ is controlled by correlation effects intrinsic to the water, it is thus no surprise that the (oscillatory) hydration forces on the mica and silica are very similar and display overall very similar trends as a function of pH and ion content. One of the most spectacular effects in this respect is the disappearance of the oscillatory hydration structure in the presence of Cs^+^ ions shown in Fig. 2. Compared to the other alkali atoms Cs^+^ and also Rb^+^ ions are less strongly hydrated (see *e.g.* ref. 73). Upon adsorption these ions tend to interact with each other leading to a steep collective adsorption isotherm *à la* Frumkin that can even lead to overscreening on mica.^14–16^ MD simulations demonstrated that this was accompanied by density depletion of water and destruction of the oscillatory density profiles.^18,34^ Similarly, Obstbaum and Sivan^74^ recently concluded from the charge regulation of silica probed by force measurements in the diffuse part of the EDL that Cs^+^ adsorbs much more strongly than Na^+^ and simultaneously expels hydration water from the surface-bound layer. In the light of these studies, we argue that the rather high density in combination with the large size of adsorbed Cs^+^ (and Rb^+^) ions then eventually reduces the direct access of too many water molecules to the solid surface and thereby alters the structure of the first surface-bound hydration layer to which the rest of the surface hydration structure is anchored. In fact, some simulation studies indeed point to a loss and even reversal of the preferred orientation of the water dipole directly at the interface.^75,76^

### Monotonically decaying hydration forces and the polarization of water

**Fig. 7:**
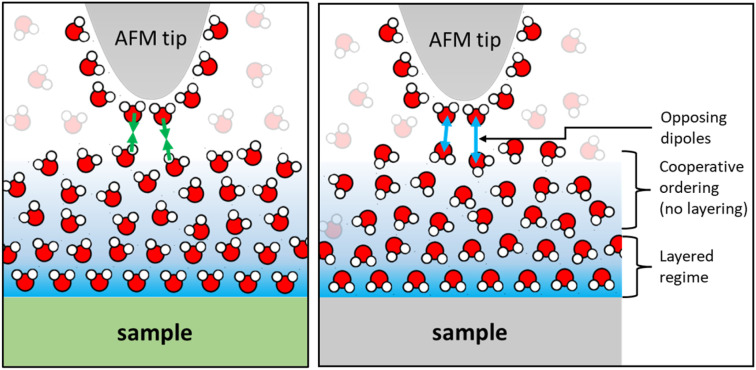
Schematic of water layers with highly ordered surface bound layers of opposite polarity and gradually increasing positional and orientational order with increasing distance from the solid surface.

Finally, we would like to comment on the at first glance perhaps less spectacular but nevertheless manifest reversal of the monotonically decaying force from repulsive for small alkali cations to attractive for Cs^+^ (Fig. 2) and for divalent Ca^2+^ ions (Fig. 4) on mica. As discussed before, the attractive monotonic short-range force can neither be attributed to van der Waals attraction nor to ion–ion correlations, which are primarily expected for higher salt concentrations and multivalent ions^10–16^ and is instead interpreted as the monotonic part of a hydration force. A qualitative explanation of such a monotonically decaying hydration force was first proposed in a phenomenological model by Marčelja and Radić.^77^ They proposed a Ginzburg–Landau type energy functional in terms of an order parameter field that describes the orientation of the water molecules. Using the lowest order approximation with a gradient-square term in the energy functional gives exponentially decaying order parameter fields and eventually to equally exponentially decaying forces. The forces can be repulsive or attractive, depending on whether the boundary conditions, *i.e.* the anchoring conditions for the order parameter field are symmetric or antisymmetric. For two identical surfaces approaching each other (including the typical mica–mica configuration in SFA experiments), the model thus always predicts repulsive forces. However, if the water dipole moments in the surface-bound layers are oppositely oriented, the model predicts exponentially decaying attractive hydration force. In the present experiments we are obviously dealing with a sharp tip above a surface rather than two flat surfaces facing each other. Assuming that the hydration state of our ‘solvent tip’ is not affected by the fluid composition, we can nevertheless expect that our ultra-sharp AFM tips are sensitive to the hydration structure of the substrate, as illustrated in Fig. 7. For pure water and in the presence of small cations that do not significantly affect the hydration structure of the substrate, we therefore obtain a more or less pronounced exponentially decaying repulsive hydration force superimposed onto the oscillatory contribution. The fact that adsorbed Cs^+^ and Ca^2+^ reverse the sign and lead to a monotonically decaying attractive hydration force, as shown in Fig. 2 and 4, then indicates that these adsorbed ions flip the average orientation of water molecules in the surface-bound layer at one of the interacting surfaces. This interpretation is consistent with some molecular simulations that report a reversal of the average orientation of water dipoles for adsorbed divalent ions (Ca^2+^ and Mg^2+^) and for Cs^+^.^75,76^

We note that the original model by Marčelja and Radić^77^ has been extended significantly throughout the decades. It has been connected to the non-local dielectric response of polar liquids providing a direct link between the phenomenological order parameter and the polarization density of the fluid.^78–80^ Moreover, the integration of additional contributions to the polarization with a second polarization field as well as higher order terms in the free energy functional have led to equations that provide a consistent description of both oscillatory and monotonically decaying hydration forces within a single model. The sensitivity of AFM spectroscopy measurements to subtle changes of the surface hydration structure may provide interesting opportunities to test such models in dedicated experiments in the future.

## Conclusion

Our AFM measurements demonstrate that both atomically smooth, crystalline mica surfaces and somewhat rougher amorphous silica surfaces display robust oscillatory and monotonically decaying hydration forces if probed by a sufficiently sharp tip. Measurements with AFM tips of variable size ranging from ≈2 nm to 250 nm demonstrate that the frequently reported absence of oscillatory hydration forces on silica does not reflect an intrinsic property of the silica–water interface but arises merely from an averaging effect for tip radii exceeding the characteristic lateral scale of the surface roughness, even if roughness amplitude does not exceed the size of a water molecule. On both substrates, oscillatory hydration forces are found for pure water and in the presence of all cations – with the exception of Cs^+^ – irrespective of their concentration and the solution pH. This suggests that hydration force oscillations should be considered an intrinsic property of the solid–water interfaces, in contrast to classical SFA studies that reported force oscillations only beyond some minimum salt concentration. For all measurements with sufficiently sharp tips, the characteristic spacing of force maxima is consistent with the diameter of a water molecule, independent of the salt concentration. No indications of spacings corresponding to hydrated ion diameters or ion pairs are found, consistent with our interpretation that the forces reflect an intrinsic property of water at these hydrophilic interfaces. Furthermore, the similarity between hydration forces on mica and silica and their almost negligible dependence on pH suggest that the hydration forces hardly depend on the surface charge.

The summary of all these observations suggests that the hydration forces are dominated by the direct interaction of the mica and silica substrates with water and by the propagation of this information *via* water–water correlations from the interface into the fluid. Ions are not needed to generate oscillatory hydration forces and affect them only weakly for most of the cases studied here. Only the strongly adsorbing weakly hydrated Cs^+^ ions disrupt the intrinsic hydrogen bonding network to an extent that breaks down the oscillatory hydration structure and reverses (on mica) the sign of the monotonic hydration force. Divalent Ca^2+^ and Mg^2+^ ions also induce attractive monotonic hydration forces, presumably because they reverse the orientation of water dipoles at the interface. The detection of monotonically decaying hydration forces with system-dependent repulsive and attractive character may offer a new pathway to characterize interfacial polarization of water by atomic force microscopy.

## Conflicts of interest

There are no conflicts to declare.

## Supplementary Material

FD-246-D3FD00049D-s001
