# Supplementary material for: Ion adsorption and hydration forces: a comparison of crystalline mica vs. amorphous silica surfaces
Source: Faraday Discuss. 2023 Mar 17;246:274–95. doi: 10.1039/d3fd00049d (PMC10568262; doi:10.1039/d3fd00049d)
Supplement: FD-246-D3FD00049D-s001 [file FD-246-D3FD00049D-s001.pdf]

Electronic Supplementary Material (ESI) for Faraday Discussions.
This journal is © The Royal Society of Chemistry 2023

Supporting Information

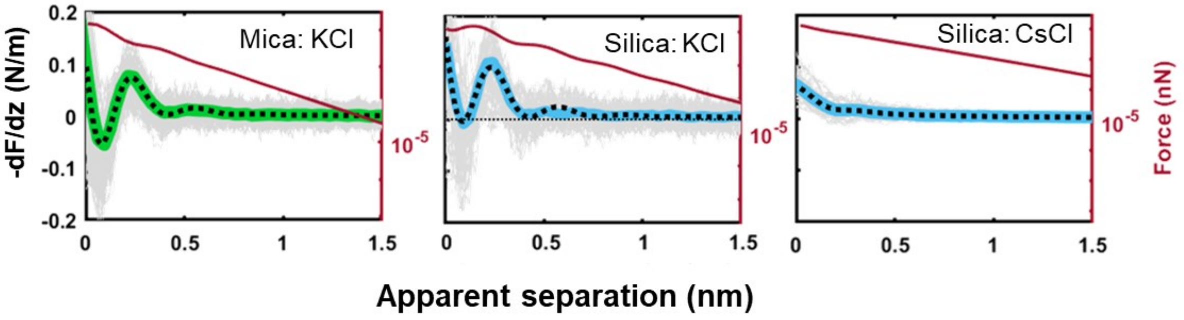

Figure SI1. Averaged force gradient ($-dF/dz$; thick green lines for mica and thick blue lines for silica, respectively) and semi-log plot of force (F thin dark red lines) versus apparent tip-sample separation measured in in various salts at a fixed concentration of 50 mM. All the data were recorded with the same AFM tip at pH 6. Dotted lines: empirical fits to equation (1).

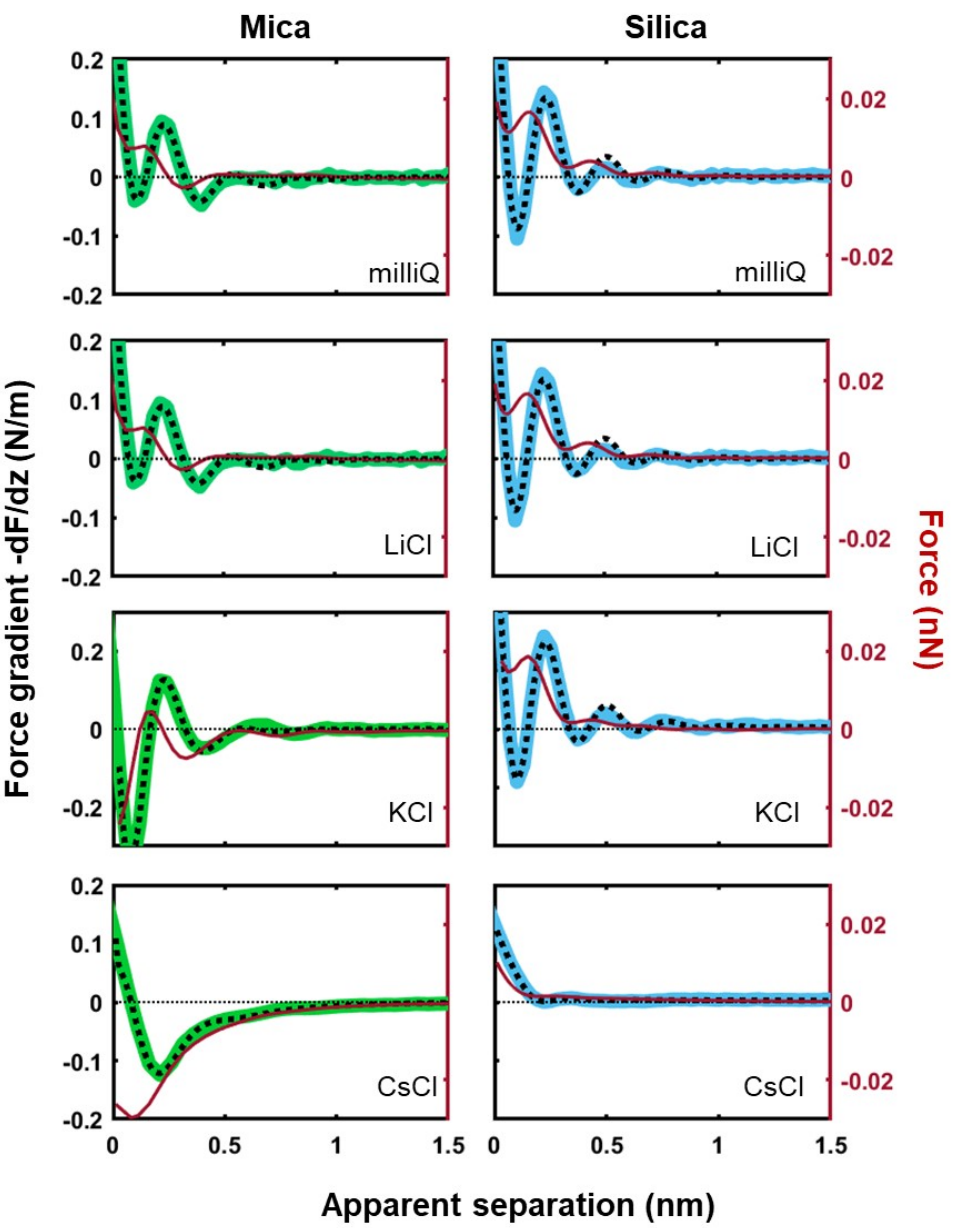

Figure SI2. Averaged force gradient ($-dF/dz$; thick green lines for mica and thick blue lines for silica, respectively) and force (F thin dark red lines) versus apparent tip-sample separation measured in pure water (light grey: 180 individual force curves) and in various salts at a fixed concentration of 50 mM. All the data were recorded with the same AFM tip at pH 6. Dotted lines: empirical fits to equation (1).

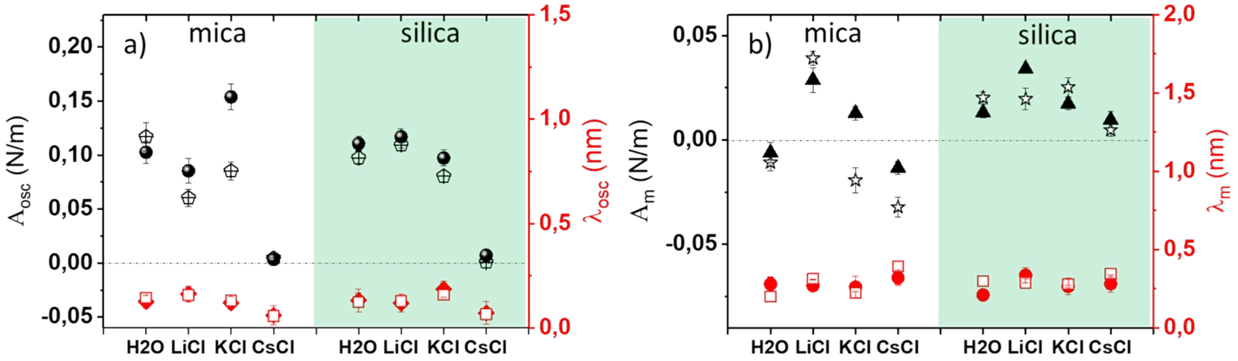

Figure SI3. Fit coefficients indicating the strength and decay length of a) the monotonic and b) the oscillatory contribution to the hydration force (data from Figure 2 and SI2). The left axis of figures indicates the magnitude and the right axis shows the decay length of the hydration forces.

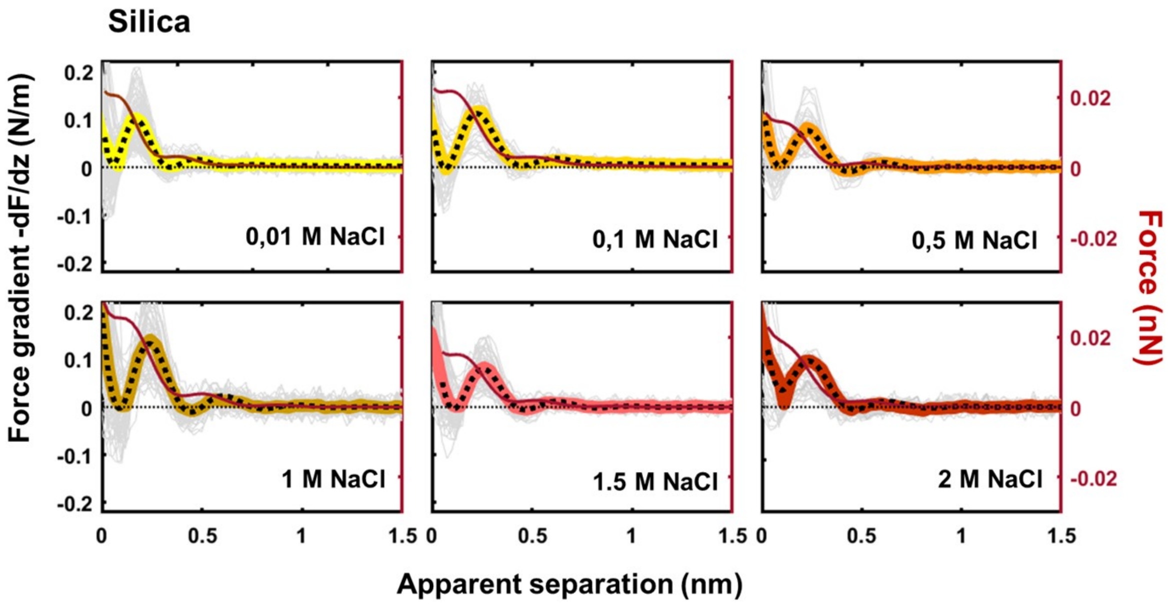

Figure SI4. Averaged force gradient ($-dF/dz$; thick colored lines) and force (F thin dark read lines) versus apparent tip-sample separation measured between silica and amorphous silica surfaces in various NaCl concentrations (light grey: 50 individual force curves). All the data were recorded with the same AFM tip at pH 6. Dotted lines: empirical fits to equation (1).

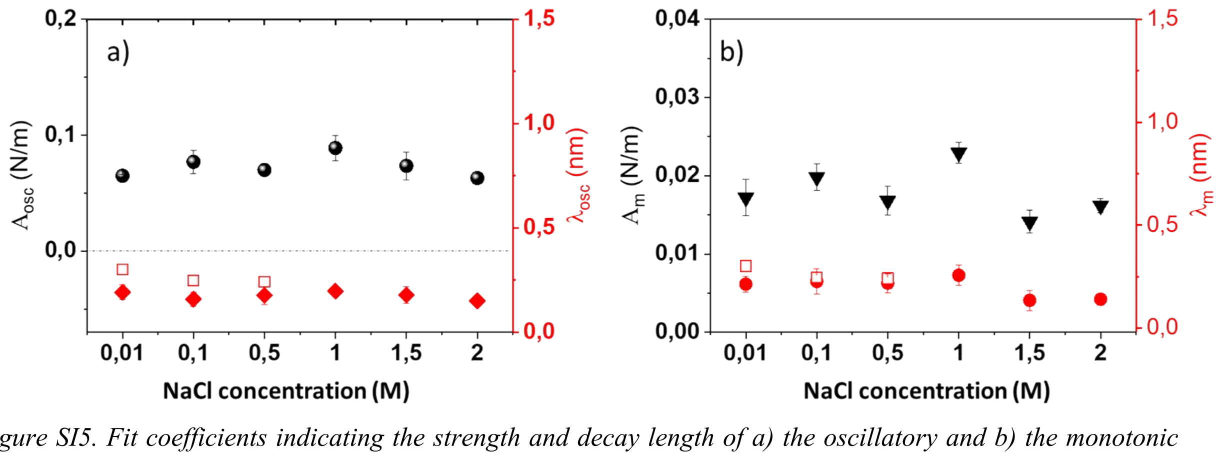

Figure SI5. Fit coefficients indicating the strength and decay length of a) the oscillatory and b) the monotonic contribution to the hydration force (data from Figure 3). The left axis of figures indicates the magnitude and the right axis shows the decay length of the monotonic part of the hydration force.

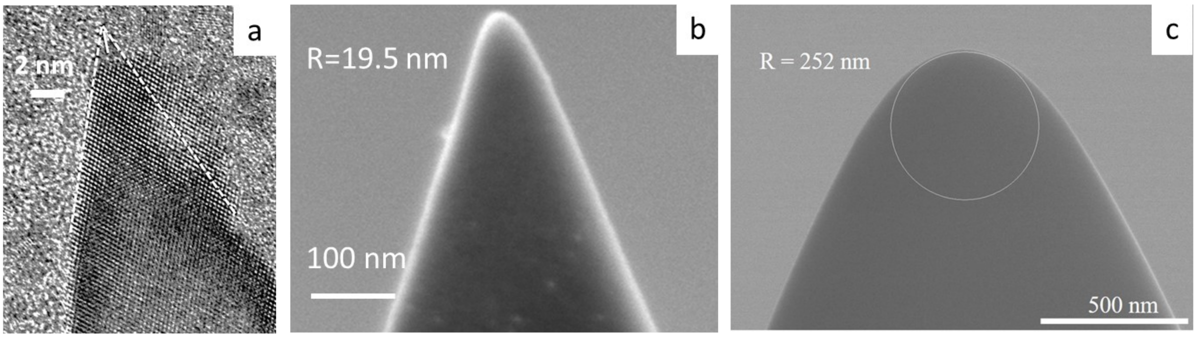

Figure SI6. HR-TEM and SEM images of the AFM tips used for data displayed in Figure 5. a) HR-TEM images of silicon-based ArrowUHFAuD. The crystalline silicon core is clearly visible, as is a layer of amorphous silicon oxide embedded in amorphous carbon matrix. b) SEM image of silicon probe (MikroMash NSC36/Cr-Au BS) covered by a 1–2 nm thick native oxide layer. c) SEM image of a hemispherical colloidal AFM probe (LRCH, Team Nanotec) made of silicon with a thin native oxide layer having a nominal tip radius of 250 nm. The radius of the AFM tip is obtained by fitting a circle to the hemispherical tip.
